# Serum Sex Hormone Binding Globulin Concentration as a Predictor of Ovarian Response During Controlled Ovarian Hyperstimulation

**DOI:** 10.3389/fmed.2021.719818

**Published:** 2021-11-04

**Authors:** Junyu Zhai, Shang Li, Yinci Zhu, Yun Sun, Zi-Jiang Chen, Yanzhi Du

**Affiliations:** ^1^Center for Reproductive Medicine, Ren Ji Hospital, School of Medicine, Shanghai Jiao Tong University, Shanghai, China; ^2^Shanghai Key Laboratory for Assisted Reproduction and Reproductive Genetics, Shanghai, China; ^3^Center for Reproductive Medicine, Cheeloo College of Medicine, Shandong University, Jinan, China; ^4^National Research Center for Assisted Reproductive Technology and Reproductive Genetics, Shandong University, Jinan, China; ^5^Key Laboratory of Reproductive Endocrinology of Ministry of Education, Shandong University, Jinan, China

**Keywords:** controlled ovarian hyperstimulation, ovarian response, SHBG, polycystic ovary syndrome, *in vitro* fertilization

## Abstract

**Purpose:** Serum concentrations of sex hormone binding globulin (SHBG), a glycated homodimeric plasma transport protein, correlate positively with the total number of follicles in women with infertility. However, the relationship between serum SHBG concentrations and the ovarian response during controlled ovarian hyperstimulation (COH) and whether this relationship differs between women with and without polycystic ovary syndrome (PCOS) remains unclear.

**Methods:** The study cohort included 120 participants (60 non-PCOS and 60 PCOS) undergoing *in vitro* fertilization. Serum samples were collected from each participant every 2–3 days during the COH cycle. The concentrations of serum SHBG and other sex hormones were determined to investigate the relationship between serum SHBG concentrations and the ovarian response in women with and without PCOS.

**Results:** We found that the serum SHBG concentration was positively correlated with the ovarian response in non-PCOS patients but not in PCOS patients.

**Conclusion:** The serum SHBG concentration may be clinically useful as a predictor of the ovarian response during COH in patients without PCOS.

## Introduction

Controlled ovarian hyperstimulation (COH) is critical for follicle development and oocyte retrieval for *in vitro* fertilization (IVF). Considering that the ovarian response varies between individuals and is affected by multiple factors, individualization of treatment protocols can improve outcomes in women with poor ovarian response and reduce risk of ovarian hyperstimulation in women with excessive ovarian response (1). For example, women with obesity typically need larger doses of gonadotropin (2), whereas those with polycystic ovary syndrome (PCOS) have a strong response to ovarian stimulation (3). Therefore, prediction and observation of the ovarian response during the COH cycle are of clinical importance. Serum indicators including 17β-estradiol (E_2_), anti-müllerian hormone (AMH), and inhibin B have been suggested as predictors of the ovarian response during COH (4, 5).

Sex-hormone–binding globulin (SHBG) is a glycated homodimeric plasma transport protein mainly synthesized in the liver. SHBG is known to regulate the concentrations of circulating androgens and estrogens by binding to them (6). In addition, locally produced, membrane-bound SHBG exerts direct effects on the cellular uptake of sex steroids and cell proliferation in hormone-responsive tissues through the activation of a specific high-affinity receptor present in the plasma membrane (7). Such direct effects of SHBG occur in the ovaries, corpora lutea, and luteinized granulosa cells (8). Therefore, it is implied that SHBG is involved in sex hormone regulation and female reproductive physiology. A study previously reported a positive correlation between SHBG concentrations and the total number of follicles in women undergoing IVF for tubal and/or male-factor infertility (9). Moreover, the SHBG rs6259 polymorphism correlates with the outcomes of IVF treatment (10). This evidence further suggests that SHBG plays a role in ovarian follicle development and IVF outcomes, and it raises the question of whether serum SHBG concentrations correlate with the degree of ovarian response during COH.

PCOS, a common endocrine disorder among reproductive-aged women, is characterized by anovulation, infertility, and hyperandrogenism. Hyperandrogenism and insulin resistance concurrently contribute to PCOS pathogenesis through mechanisms that remain unclear. Most patients with PCOS are at a high risk for type 2 diabetes and cardiovascular disease (CVD) (11), and serum SHBG concentrations are reported to be altered in PCOS, metabolic syndrome, type 2 diabetes, and CVD (12, 13). Specifically, overweight or obese women with PCOS have decreased serum SHBG concentrations and increased serum total testosterone concentrations and free androgen index, indicating that SHBG abnormalities are involved in PCOS pathophysiology. Recent studies stress the importance of SHBG measurement in the diagnosis and management of PCOS (14, 15). However, the correlation between serum SHBG concentrations and the ovarian response in PCOS patients remains unclear and warrants further investigation.

To identify a new indicator of the ovarian response for clinical applications, the present study aimed to determine whether serum SHBG concentrations correlate with the ovarian response and COH outcomes. We investigated the basal concentration and changes in serum SHBG concentrations during the COH cycle induced by the gonadotropin-releasing hormone (GnRH) antagonist protocol and compared the findings between subjects with and without PCOS.

## Materials and Methods

### Study Participants

Blood samples were collected from 120 women who underwent IVF at the reproductive medical center of Ren Ji Hospital from August 1, 2018 to October 1, 2019. All enrolled participants met the following requirements: age, 20–35 years; undergoing the first cycle of IVF; presence of primary or secondary infertility; and body mass index (BMI) of 16–33 kg/m^2^. Patients diagnosed with endometriosis, premature ovarian failure, abnormal thyroid function, or previous ovarian trauma caused by surgery were excluded. Following the selection process, all participants underwent a full infertility workup that included a basal pelvic ultrasound, assessment of ovarian and thyroid hormones, and serological test for HIV, hepatitis B, and hepatitis C. Among the 120 participants who were enrolled in this study, 60 were diagnosed with PCOS according to Rotterdam criteria, including oligomenorrhea or amenorrheas combined with either hyperandrogenism or polycystic ovaries by B ultrasound in menstrual period. Polycystic ovaries were defined as the presence of an ovary containing 12 or more antral follicles measuring 2–9 mm in diameter. Other causes of hyperandrogenism such as tumors, congenital adrenal hyperplasia, hyperprolactinemia were ruled out (16). The non-PCOS participants (*n* = 60) were undergoing IVF for tubal or male-factor infertility.

### Ovarian Stimulation and Clinical Pregnancy

The GnRH antagonist protocol is widely used clinically because of its strong controllability and few complications, especially in PCOS patients (17). All enrolled participants received the GnRH antagonist protocol for COH to eliminate treatment protocol as a variable. Recombinant follicle stimulating hormone (rFSH) was used to initiate COH, and the results of B-mode ultrasound imaging and serum hormone concentrations guided the clinicians' decisions on the timing and dosage of gonadotropin (Gn) (Gonal F; EMD-Serono, MA, USA). GnRH antagonist (Cetrotide, Merck, NJ, USA) was used when largest follicle exceeded 12 mm. Human chorionic gonadotropin (hCG) (Livzon, Guangdong, China) was administered to induce oocyte maturation and ovulation when at least two lead follicles have reached ≥ 1.8 cm and serum E_2_ level match the size and numbers of lead follicles. Oocytes were retrieved transvaginally 34–36 h after hCG administration. Clinical pregnancy was defined as the presence of a gestational sac in the uterine cavity at 28–35 days after embryo transfer, as detected on ultrasonography.

### Sample Collection

All participants received basal ovarian reserve testing (assessment of sex hormones and AMH on day-2 of the period). Patients also underwent an antecubital venipuncture blood draw every 2–3 days from the beginning of Gn treatment to assess the serum concentrations of FSH, luteinizing hormone (LH), E_2_, and progesterone (P_4_). Serum samples were collected to assess the SHBG concentration. A total of 480 blood samples were collected. During COH, B-mode ultrasound was used to detect follicles and determine the endometrial thickness until the day of hCG administration.

### Hormone Concentration Measurements

Serum samples collected during COH were assayed for FSH, LH, E_2_, and P_4_ using the Roche Electrical Chemiluminescence Immunoassay (Roche, Basel, Switzerland) and all the 480 samples were tested. Serum SHBG concentrations were assayed using commercially available enzyme linked immunosorbent assay kits (R&D systems, MN, USA). All assays were carried out according to standard protocols by the same experienced technician to minimize the effect of interassay variability.

### Statistical Analysis

Spearman's correlation coefficient was used to describe the non-parametric measure of dependence between different variables in COH and the number of retrieved oocytes and embryos. The correlation between serum SHBG and other indicators during COH was also analyzed using Spearman's correlation. Student's *T*-test was used to compare the PCOS group to the non-PCOS group and the normoresponders to the high responders. Changes in serum SHBG after Gn treatment as compared to baseline values were analyzed using the paired-sample *T*-test. The power to discriminate normoresponders from high responders was evaluated by receiver operating characteristic (ROC) curve analysis. Sensitivity, specificity, and the area under the ROC curve (AUC-ROC) were obtained for each model. The 95% confidence intervals were calculated for each of the estimates. The pregnancy rate and live birth rate was analyzed using Pearson's chi-square test. SPSS software (IBM Corp., NY, USA) was used for all analyses. Significance was defined as *P* < 0.05, and all results are expressed as the mean ± standard deviation.

## Results

### Baseline Characteristics of All Participants

The demographic and baseline characteristics of the cohort are shown in Table 1. Mean age and BMI were 29.65 years and 22.51 kg/m^2^, respectively. The mean basal concentrations of FSH, LH, E_2_, P_4_, and AMH were 6.62 IU/L, 6.59 IU/L, 48.74 pg/mL, 0.22 ng/mL, and 6.98 ng/mL, respectively. Moreover, we also detected the basal SHBG level, which means the SHBG levels of D2 in menstrual cycle. Mean basal SHBG was 162.17 nmol/L in the total cohort, including PCOS and non-PCOS subgroups. The mean dose of Gn used during COH was 1491.02 IU, and, on average, 17.09 oocytes and 6.35 embryos were obtained from each patient.

**Table 1:** Clinic characteristics of non-PCOS and PCOS subgroups.

	**All participants**	**non-PCOS**	**PCOS**	***P***
	**(*n* = 120)**	**(*n* = 60)**	**(*n* = 60)**	
Age (years)	29.65 ± 3.21	30.29 ± 3.30	29.02 ± 3.03	0.061
BMI (kg/m^2^)	22.51 ± 3.58	22.38 ± 3.74	22.64 ± 3.46	0.726
Basal FSH (IU/L)	6.62 ± 1.65	7.03 ± 1.80	6.22 ± 1.39	0.019
Basal LH (IU/L)	6.59 ± 2.98	5.67 ± 2.97	7.51 ± 2.71	0.003
Basal E_2_ (pg/mL)	48.74 ± 50.85	48.69 ± 51.58	48.80 ± 50.70	0.992
Basal P_4_ (ng/mL)	0.22 ± 0.20	0.24 ± 0.26	0.19 ± 0.10	0.232
Basal AMH (ng/mL)	6.98 ± 3.71	4.89 ± 2.30	9.13 ± 3.67	0.000
Total testosterone (nmol/L)	1.30 ± 0.67	0.87 ± 0.49	1.72 ± 0.82	0.031
HOMA-IR	1.95 ± 0.63	1.21 ± 0.34	2.68 ± 0.82	0.018
Gn (IU)	1491.02 ± 355.442	1516.75 ± 320.03	1465.98 ± 390.98	0.496
Number of retrieved oocytes	17.09 ± 8.47	13.40 ± 6.07	20.78 ± 8.97	0.000
Number of embryos	6.35 ± 3.89	5.11 ± 3.47	7.61 ± 3.93	0.002
Endometrium on hCG day (mm)	8.72 ± 2.37	8.86 ± 2.60	8.59 ± 2.14	0.621
E_2_ on hCG day (pg/mL)	3121.03 ± 2081.57	2297.23 ± 1456.30	3926.53 ± 2291.52	0.000
Basal SHBG (nmol/L)	162.17 ± 90.15	155.05 ± 100.43	169.61 ± 78.44	0.440
SHBG on hCG day (nmol/L)	128.49 ± 66.19	119.88 ± 69.07	137.50 ± 62.53	0.204
Pregnancy rate	0.60	0.62	0.58	0.709
Live birth rate	0.49	0.48	0.50	0.855
ΔE_2_	2989.33 ± 1999.84	2239.13 ± 1410.37	3739.53 ± 2224.70	0.0002
ΔLH	−4.06 ± 3.13	−3.05 ± 3.28	−5.077 ± 2.64	0.1117
ΔP_4_	0.45 ± 0.39	0.36 ± 0.37	0.53 ± 0.41	0.045

*ΔE_2_: E_2_ on hCG day – basal E_2_*.

*ΔLH: LH on hCG day – basal LH*.

*ΔP_4_: P_4_ on hCG day – basal P_4_*.

*Pregnancy rate: the pregnancy rate after first embryo transfer cycle*.

*Liver birth rate: accumulated live birth rate within 1 year after oocyte retrieval*.

### Changes in Serum SHBG Concentrations During COH in the Total Cohort

**Figure 1:**
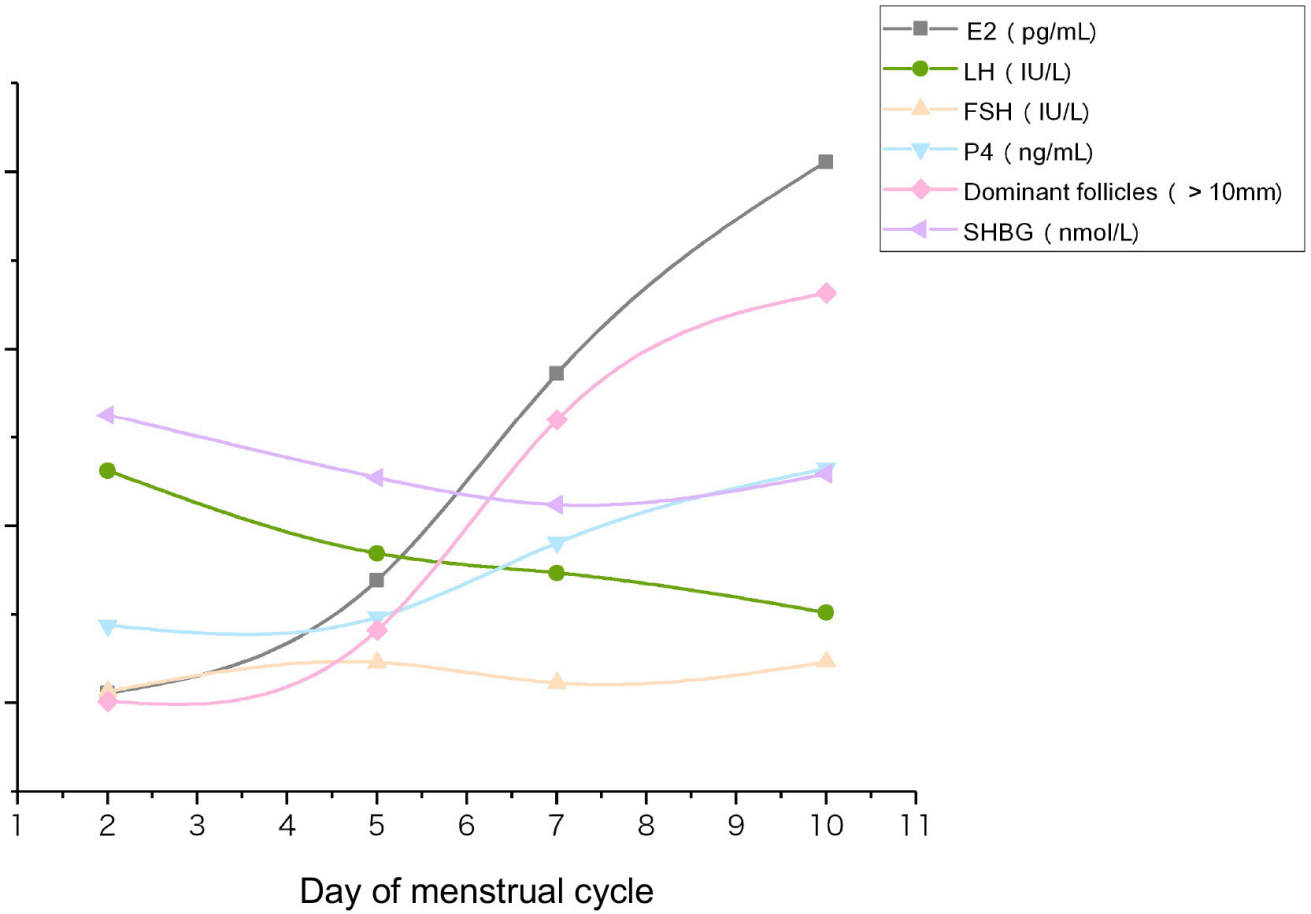
Changes in serum concentrations of follicle-development–related hormones, SHBG, and the number of dominant follicles during COH. Serum samples from each participant were collected from the beginning of Gn (D2) to hCG administration day (D10). Data were averaged for the total cohort (*n* = 120).

Analysis of all 120 participants together, including PCOS and non-PCOS groups, revealed an overall decline in serum SHBG concentrations during COH (*P* = 0.000). Serum E_2_ concentrations and the number of dominant follicles (diameter > 10 mm) increased significantly, whereas the serum LH decreased (Figure 1). No difference was observed in the variation trend between the PCOS and non-PCOS groups (Supplementary Figure 1). SHBG level decreased from basal 162.17 nmol/L to 128.49 nmol/L on hCG day after Gn treatment, exhibiting the same trend as shown in Figure 1.

### Correlation of Serum SHBG Concentrations With Hormone Concentrations and the Number of Retrieved Oocytes and Embryos During COH in the Total Cohort

**Figure 2:**
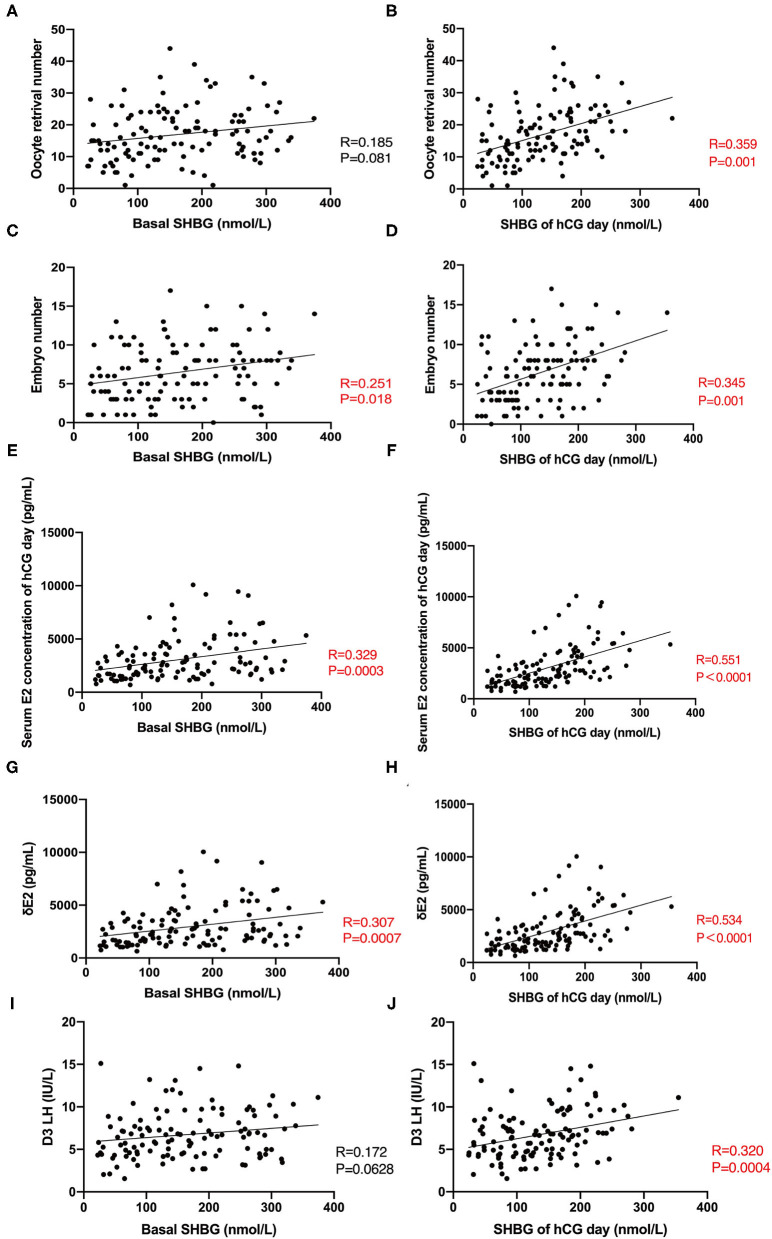
Correlation between basal SHBG, hCG-day SHBG, and clinic indicators during COH. **(A,B)** Correlation between basal SHBG concentration, hCG-day SHBG concentration, and number of retrieved oocytes. **(C,D)** Correlation between serum basal SHBG concentration, hCG-day SHBG concentration, and number of embryos. **(E,F)** Correlation between serum basal SHBG concentration, hCG-day SHBG concentration, and serum E_2_ concentration on hCG day. **(G,H)** Correlation between serum basal SHBG concentration, hCG-day SHBG concentration, and ΔE_2_. **(I,J)** Correlation between serum basal SHBG concentration, hCG-day SHBG concentration, and basal LH concentration.

The number of retrieved oocytes and embryos was the primary outcome variable representing the ovarian response during COH. We observed that the serum SHBG concentration on hCG day correlated positively to the number of retrieved oocytes and embryos (Figures 2A–D), suggesting that serum SHBG may be predictive of the ovarian response to the GnRH antagonist protocol. However, ΔSHBG (SHBG concentration on hCG day minus the basal SHBG concentration) did not correlate with the ovarian response variables in COH. The ovarian response also correlated positively with the basal serum concentration of AMH, E_2_ on hCG day, ΔE_2_ (E_2_ concentration on hCG day minus the basal E_2_ concentration), and ΔP_4_ (P_4_ concentration on hCG day minus the basal P_4_ concentration) and correlated negatively with ΔLH (LH concentration on hCG day minus the basal LH concentration) and basal FSH concentration (Table 2). Therefore, serum SHBG, basal AMH, basal FSH, ΔE_2_, ΔP_4_, and ΔLH could be used to predict the ovarian response during the COH cycle.

**Table 2:** Correlation between hormone concentrations during COH and the number of retrieved oocytes and embryos in the total cohort.

	**Number of**	**Number of**
	**oocytes retrieved**	**embryos**
Basal SHBG (nmol/L)	*R* = 0.185	*R* = 0.251
	*P* = 0.081	*P* = 0.018
SHBG on hCG day (nmol/L)	*R* = 0.359	*R* = 0.345
	*P* = 0.001	*P* = 0.001
Basal AMH (ng/mL)	*R* = 0.529	*R* = 0.401
	*P* = 0.000	*P* = 0.000
E_2_ on hCG day (pg/mL)	*R* = 0.64	*R* = 0.542
	*P* = 0.000	*P* = 0.000
ΔE_2_ (pg/mL)	*R* = 0.636	*R* = 0.533
	*P* = 0.000	*P* = 0.000
Basal LH (IU/L)	*R* = 0.289	*R* = 0.220
	*P* = 0.006	*P* = 0.038
ΔLH (IU/L)	*R* = −0.316	*R* = −0.335
	*P* = 0.000	*P* = 0.000
Basal FSH (IU/L)	*R* = −0.425	*R* = −0.197
	*P* = 0.000	*P* = 0.064
ΔP_4_ (ng/mL)	*R* = 0.446	*R* = 0.256
	*P* = 0.000	*P* = 0.016

*n = 120*.

In subsequent investigations of the relationship between serum SHBG and hormone concentrations during COH in the total cohort, we observed that serum SHBG concentrations (both basal and hCG-day concentrations) correlated positively with the serum E_2_ concentration on hCG day and ΔE_2_ (Figures 2E–H) while only hCG-day SHBG correlated with D3 LH (Figures 2I,J).

### Serum SHBG and AMH Concentrations in Normo- and High-Responder Subgroups

As a recognized predictor of ovarian reserve and ovarian response, the basal AMH concentration was significantly higher in the high responders than in the normoresponders (*P* = 0.000) (18). Furthermore, in the total cohort, high responders had significantly higher basal and hCG-day serum SHBG concentrations (*P* = 0.035 and 0.003, respectively) (Table 3).

**Table 3:** Serum SHBG and AMH concentrations in normo- and high-responder subgroups.

	**Normoresponders (4–15 oocytes) *N* = 61**	**High responders (>15 oocytes)*N* = 59**	***P***
Basal SHBG (nmol/L)	142.65 ± 96.23	182.48 ± 78.77	0.035
SHBG on hCG day (nmol/L)	108.44 ± 59.45	149.31 ± 66.30	0.003
Basal AMH (ng/mL)	5.76 ± 2.90	8.25 ± 3.83	0.000

### Serum SHBG Concentrations in PCOS and Non-PCOS Subgroups

To clarify the role of serum SHBG concentration during COH in PCOS, we investigated the basic characteristics and potential predictors in PCOS and non-PCOS subgroups (Table 1). We observed significantly difference in basal AMH, FSH, LH, HOMA-IR, number of oocytes and embryos between PCOS vs. non-PCOS subgroup without any changes in BMI (Table 1). Moreover, basal AMH, hCG-day E_2_ concentration, ΔE_2_, ΔP_4_, and basal FSH concentrations correlated with the number of oocytes retrieved in both the PCOS and non-PCOS subgroups, whereas the hCG-day SHBG concentration correlated positively with the number of oocytes retrieved only in the non-PCOS participants (Table 4). We observed significantly higher serum SHBG concentrations in high responders than in the normoresponders only in the non-PCOS subgroup (basal concentration, P = 0.014; hCG day, 0.011) (Table 5). Therefore, the serum SHBG concentration could be used as a predictor of follicle development during COH only for non-PCOS participants.

**Table 4:** Correlation between hormone concentrations and the number of oocytes retrieved in non-PCOS and PCOS subgroups.

	**Non-PCOS**	**PCOS**
Basal SHBG (nmol/L)	*R* = 0.245	*R* = 0.124
	*P* = 0.105	*P* = 0.416
SHBG on hCG day (nmol/L)	*R* = 0.386	*R* = 0.238
	*P* = 0.009	*P* = 0.118
Basal AMH (ng/mL)	*R* = 0.307	*R* = 0.406
	*P* = 0.050	*P* = 0.010
E_2_ on hCG day (pg/mL)	*R* = 0.543	*R* = 0.556
	*P* = 0.000	*P* = 0.000
ΔE_2_ (pg/mL)	*R* = 0.572	*R* = 0.535
	*P* = 0.000	*P* = 0.000
Basal LH (IU/L)	*R* = 0.091	*R* = 0.151
	*P* = 0.551	*P* = 0.323
ΔLH (IU/L)	*R* = −0.221	*R* = −0.185
	*P* = 0.144	*P* = 0.224
Basal FSH (IU/L)	*R* = −0.419	*R* = −0.352
	*P* = 0.004	*P* = 0.018
ΔP_4_ (ng/mL)	*R* = 0.382	*R* = 0.482
	*P* = 0.010	*P* = 0.001

**Table 5:** Comparison of serum SHBG concentrations according to ovarian response in non-PCOS and PCOS subgroups.

	**Non-PCOS (normoresponders vs. high responders)**	**PCOS (normoresponders vs. high responders)**
Basal SHBG (nmol/L)	*P* = 0.014	*P* = 0.878
SHBG on hCG day (nmol/L)	*P* = 0.011	*P* = 0.252

**Figure 3:**
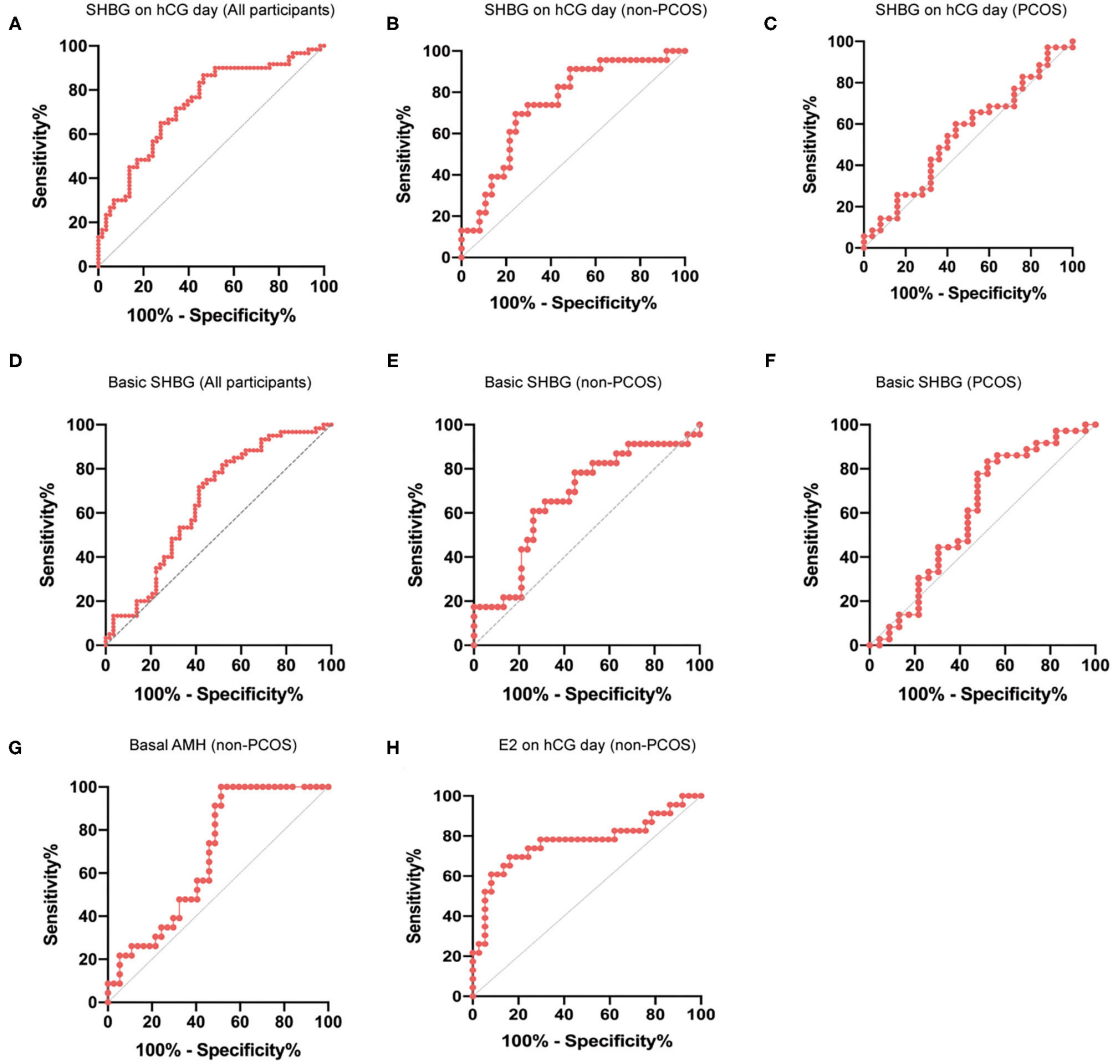
ROC curve for prediction of ovarian response using hCG-day SHBG. **(A)** ROC curve for prediction of ovarian response (normal response and high response) with hCG-day SHBG in the total cohort (*n* = 120); *P* < 0.0001. **(B)** ROC curve for predicting ovarian response (normal response and high response) with hCG-day SHBG in non-PCOS participants (*n* = 60); *P* = 0.0015. **(C)** ROC curve for predicting ovarian response (normal response and high response) with hCG-day SHBG in PCOS participants (*n* = 60); *P* = 0.5142. **(D)** ROC curve for prediction of ovarian response (normal response and high response) with basic SHBG in the total cohort (*n* = 120); *P* = 0.0028. **(E)** ROC curve for predicting ovarian response (normal response and high response) with basic SHBG in non-PCOS participants (*n* = 60); *P* = 0.0287. **(F)** ROC curve for predicting ovarian response (normal response and high response) with basic SHBG in PCOS participants (*n* = 60); *P* = 0.1982. **(G)** ROC curve for predicting ovarian response (normal response and high response) with basal AMH in non-PCOS participants (*n* = 60); *P* = 0.018. **(H)** ROC curve for predicting ovarian response (normal response and high response) with hCG-day E_2_ in non-PCOS participants (*n* = 60); *P* = 0.0005.

ROC analysis was performed to further evaluate the value of basic and hCG-day serum SHBG concentration in predicting the ovarian response. We observed that in the total cohort, the AUCROC for serum SHBG on hCG day was greater than that for basal SHBG or ΔSHBG (Figures 3A,D; Supplementary Figure 2). ROC analysis of hCG-day SHBG in non-PCOS and PCOS subgroups showed that the AUCROC in non-PCOS and PCOS participants were 0.7450 and 0.5497, respectively (*P* = 0.0015 and 0.5142) (Figures 3B,C). Moreover, AUCROC of basic SHBG level in non-PCOS and PCOS participants were 0.6682 and 0.6014, respectively (*P* = 0.0287 and 0.1917) (Figures 3E,F). Thus, serum SHBG concentration might be a good predictor for ovarian response in patients without PCOS but not with PCOS. Finally, we compared the AUCROC for serum SHBG to that of other traditional predictors of ovarian response. We found that among the patients without PCOS, the AUCROC for basal AMH and hCG-day E_2_ was 0.6827 and 0.7697, respectively (Figures 3G,H), which were not significantly greater than those for serum SHBG concentration.

## Discussion

Our study cohort enrolled 120 participants undergoing *in vitro* fertilization and analyzed the relationship between serum SHBG and ovarian response during COH in women with and without PCOS. We found that the serum SHBG concentration was positively correlated with the ovarian response in non-PCOS patients but not in PCOS patients.

SHBG is secreted from the liver into the blood, where it binds to a variety of sex hormones. A significant negative relationship has been observed between steroid hormones and SHBG expression in the ovaries (19). Thus, SHBG is closely related to the functions of sex hormones. A previous study found that FSH receptor/SHBG/aromatase cytochrome P450 (CYP19) genotypes are associated with the ovarian response to standard Gn stimulation in women undergoing assisted reproduction (20). The SHBG concentration in follicular fluid correlates positively with the total number of follicles (9), suggesting that SHBG may be involved in follicle development and the ovarian response during COH. However, whether serum SHBG concentrations change during the COH cycle induced by using the GnRH antagonist and whether they correlate with follicular development during COH remains unknown, thereby warranting further investigation.

The findings of this study suggest that serum SHBG concentrations may predict the ovarian response during the COH cycle induced using the GnRH antagonist protocol. We observed a decrease in serum SHBG concentrations through the course of the COH cycle, showing the same trend as LH and opposing the trend in serum E_2_ concentration and number of dominant follicles. Moreover, serum SHBG concentrations correlated positively with the number of retrieved oocytes and embryos, both of which represent the ovarian response during COH. In addition, the serum SHBG concentrations of high responders were significantly higher than those of normal responders, suggesting that the serum SHBG concentration is predictive of the ovarian response.

An increase in serum SHBG concentration is reported during the course of COH induced using the GnRH agonist protocol, and a constant SHBG concentration is observed throughout the normal menstrual cycle (21). However, the decreasing trend in serum SHBG concentrations during COH observed in our study (total cohort, *P* = 0.000; non-PCOS, *P* = 0.000; PCOS, *P* = 0.001) differs from the previously reported increase (10). This inconsistency may result from differences in the COH stimulation protocols.

Plasma concentrations of proinflammatory cytokines such as tumor necrosis factor alpha (TNFα) reportedly inhibited SHBG production (22). Furthermore, SHBG may be upregulated by adiponectin through adenosine 5′-phosphate-activated protein kinase (AMPK) pathways in HepG2 cells (23), and serum SHBG concentrations may be regulated by metabolic status, such as obesity or insulin resistance. PCOS is a chronic inflammatory condition associated with increased serum TNFα (24) and decreased serum adiponectin (25) concentrations, which accompany insulin resistance and dyslipidemia. Therefore, we investigated the serum SHBG concentrations in subgroups of non-PCOS and PCOS patients. Our results show that serum SHBG concentrations did not correlate with the ovarian response in the PCOS subgroup. We observed that the PCOS participants had significantly higher serum LH, AMH, and total testosterone concentrations than the non-PCOS participants. These elevated androgen concentrations may be involved in the regulation of SHBG in PCOS. Insulin also plays a vital role in SHBG regulation. A combined analysis of 23 cross-sectional studies finds that women with type 2 diabetes have significantly lower serum SHBG concentrations than controls (26). Although an inverse relationship between serum insulin and SHBG concentrations is assumed, recent studies have seriously questioned this assumption. Investigations performed while considering more physiological conditions show that insulin does not regulate SHBG production in HepG2 cells (27). Thus, insulin may regulate SHBG, but the actual mechanism underlying such regulation is unknown. The elevated homeostatic model assessment of insulin resistance (HOMA-IR) observed in the PCOS group in our study may also contribute to the regulation of serum SHBG. In summary, the complicated metabolic, inflammatory, and hormone conditions of PCOS patients may alter SHBG regulation and disrupt the predictive role of SHBG concentrations in ovarian response during the COH cycle.

Low serum-SHBG concentration is often used as an indicator of hyperandrogenism in women with PCOS. Low SHBG is associated with obesity (11), hyperinsulinemia (28), and hyperandrogenism (29), which are commonly found in PCOS patients. Surprisingly, in this study, the basal SHBG concentrations did not differ significantly between patients with and without PCOS (Table 1). We found that the BMI did not differ between these two subgroups. BMI and liver fat have been suggested as predictors of serum SHBG concentrations (30). Patients with morbid obesity who have undergone bariatric surgery show an increase in serum SHBG concentrations, which correlates closely with weight loss (31). Furthermore, a previous study found no difference in serum SHBG concentrations between PCOS patients of normal weight and controls (32). Hence, our study results suggest that BMI might play a vital role in determining the basal SHBG concentration in PCOS patients. Obese women with PCOS are reported to have lower serum SHBG than non-obese women with PCOS, while the SHBG concentration is inversely related to the occurrence of metabolic syndrome, which also verifies our hypothesis (33).

This study has several limitations. First, given that the study focused on SHBG concentrations during COH induction, we did not investigate the relationship between serum SHBG concentrations and pregnancy outcomes. Second, to investigate the difference between serum SHBG concentrations and the ovarian response between non-PCOS and PCOS subgroups, we strictly limited the treatment protocol as well as patient age, AMH, and other variables; however, possible effects on serum SHBG concentrations caused by age, treatment protocol, or the cause of infertility cannot be ruled out.

In conclusion, the serum concentration of SHBG correlated positively with the ovarian response of patients without PCOS during COH using the GnRH antagonist protocol. However, the serum SHBG concentration is not predictive of the ovarian response in PCOS patients. This study suggests that serum SHBG concentration can be used to predict the ovarian response during COH in non-PCOS patients.

## Data Availability Statement

The original contributions presented in the study are included in the article/Supplementary Material, further inquiries can be directed to the corresponding author/s.

## Ethics Statement

The studies involving human participants were reviewed and approved by Institutional Review Board of Shanghai Jiao Tong University School of Medicine Affiliated Ren Ji Hospital. The patients/participants provided their written informed consent to participate in this study.

## Author Contributions

JZ, SL, and YD contributed to the design of the experiment, acquisition of data, and analysis and interpretation of data. YZ contributed to the collection of serum samples of PCOS and non-PCOS participants in COH cycles. JZ, SL, YS, Z-JC, and YD finished drafting the article or revised it critically for important intellectual content. YD was responsible for the final approval of the version to be published. All authors contributed to the article and approved the submitted version.

## Funding

This research was supported by grants from the National Key Research and Development Program of China (2018YFC1003202), National Natural Science Foundation (81971343 and 81901549), and Shanghai Key Laboratory for Assisted Reproduction and Reproductive Genetics (19410760300 and 20DZ2270900).

## Conflict of Interest

The authors declare that the research was conducted in the absence of any commercial or financial relationships that could be construed as a potential conflict of interest.

## Publisher's Note

All claims expressed in this article are solely those of the authors and do not necessarily represent those of their affiliated organizations, or those of the publisher, the editors and the reviewers. Any product that may be evaluated in this article, or claim that may be made by its manufacturer, is not guaranteed or endorsed by the publisher.
