# Supplementary material for: Serum Sex Hormone Binding Globulin Concentration as a Predictor of Ovarian Response During Controlled Ovarian Hyperstimulation
Source: Front Med (Lausanne). 2021 Nov 4;8:719818. doi: 10.3389/fmed.2021.719818 (PMC8600072; doi:10.3389/fmed.2021.719818)
Supplement: Supplementary file 1 [file Data_Sheet_1.docx]

**Supplementary File for**

Serum sex hormone binding globulin concentration as a predictor of ovarian response during controlled ovarian hyperstimulation


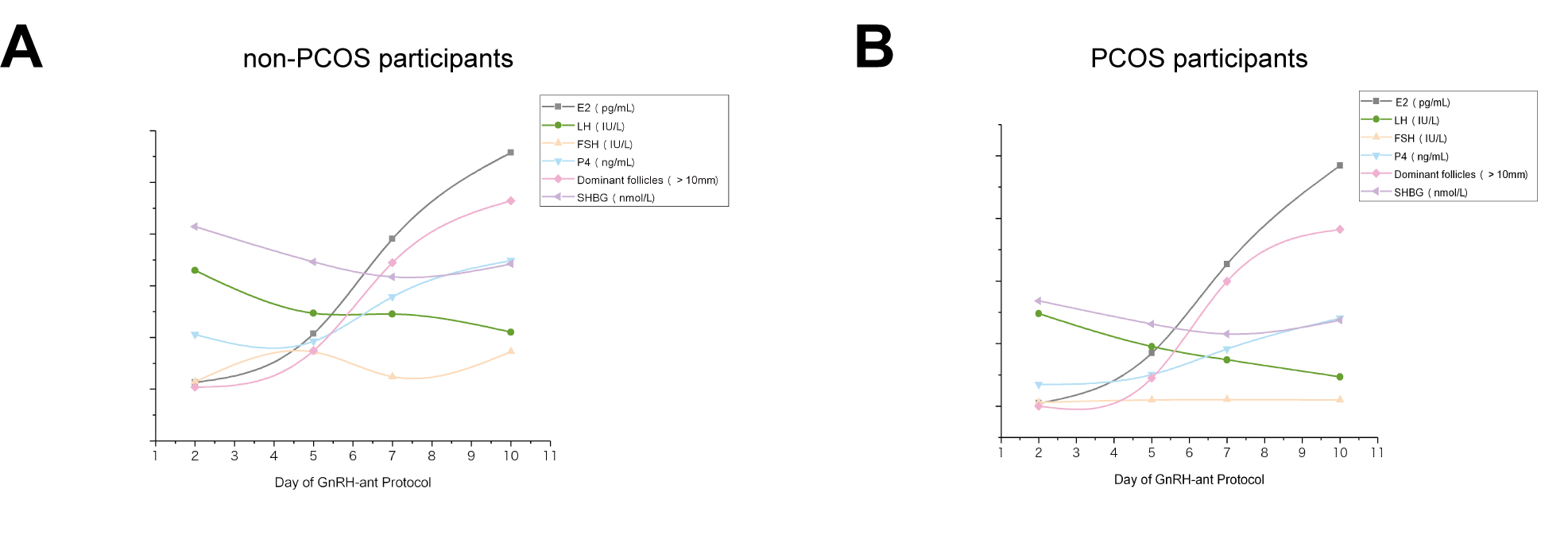


**Supplementary Figure 1. Changes in sex hormones, dominant follicle number, and serum SHBG concentrations during COH**

**(A)** non-PCOS participants (n = 60); **(B)** PCOS participants (n = 60).


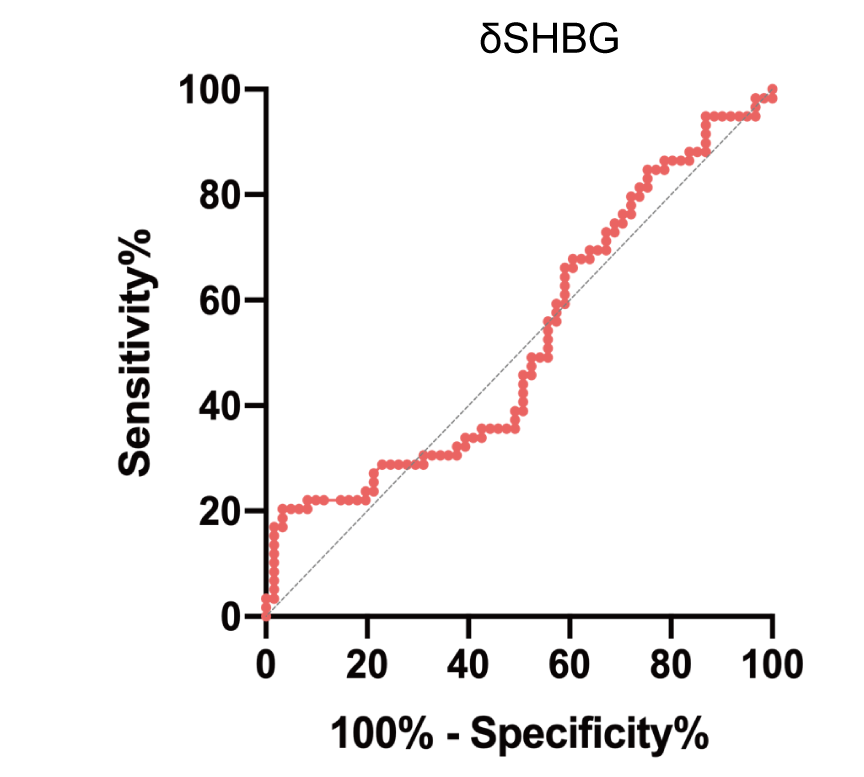


**Supplementary Figure 2.** ROC curve using ΔSHBG (hCG-day serum concentration minus basal concentration) (P = 0.6631) (n = 120).
